# A novel approach to managing biliary anastomotic stricture following orthotopic liver transplantation after failure of endoscopic retrograde cholangiopancreatography technique

**DOI:** 10.1055/a-2493-3614

**Published:** 2025-01-14

**Authors:** Baifeng Qian, Junlong Lin, Jianpeng Cai, Yunpeng Hua

**Affiliations:** 1 71068 Center of Hepato-Pancreato-Biliary Surgery, The First Affiliated Hospital of Sun Yat-sen University, Guangzhou, China

Biliary stricture is the most common complication after liver transplantation, accounting for ~40% of all biliary complications 1 2 . These complications remain a major cause of morbidity and, in severe cases, mortality. Endoscopic treatment is the first-line treatment for benign biliary stricture 3 . The success rate of endoscopic treatment of anastomotic stricture is 58%–76% in patients with living-donor liver transplantation 4 5 . Here, we show a novel approach to managing biliary anastomotic stricture following orthotopic liver transplantation after failure of the endoscopic retrograde cholangiopancreatography (ERCP) technique, even with direct cholangioscopy.

We report the case of a 54-year-old patient who presented with progressive jaundice after undergoing liver transplantation for hepatocellular carcinoma. Contrast-enhanced abdominal computed tomography revealed a biliary anastomotic stricture, with a dilated common bile duct (CBD) measuring 21 mm ( Fig. 1 ). A classic ERCP was performed, during which retrograde cholangiography revealed truncation of the CBD, with nonvisualization of the upper part of the CBD and intrahepatic bile duct. Attempts to pass through the stricture under fluoroscopy with a 0.035-inch straight guidewire were unsuccessful. Subsequent direct cholangioscopy with guidewire also failed after several attempts ( Fig. 2 ). Classic rendezvous technique of percutaneous transhepatic biliary drainage and ERCP was unsuccessful in passing through the stricture ( Fig. 3 ). Finally, percutaneous transhepatic cholangiography was performed and a rigid guidewire was inserted through the stricture under ultrasound guidance ( Fig. 4 , Video 1 ). The stricture was then dilated using a 6-mm balloon, followed by placement of a 10 × 80 mm self-expanding covered metal stent under ERCP ( Fig. 5 ).

**Fig. 1:**
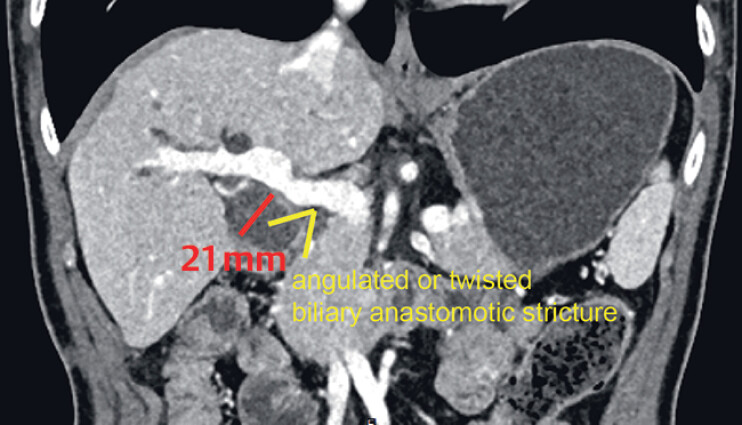
Contrast-enhanced abdominal computed tomography (coronal plane) revealed an angulated or twisted biliary anastomotic stricture, with a dilated common bile duct measuring 21 mm.

**Fig. 2:**
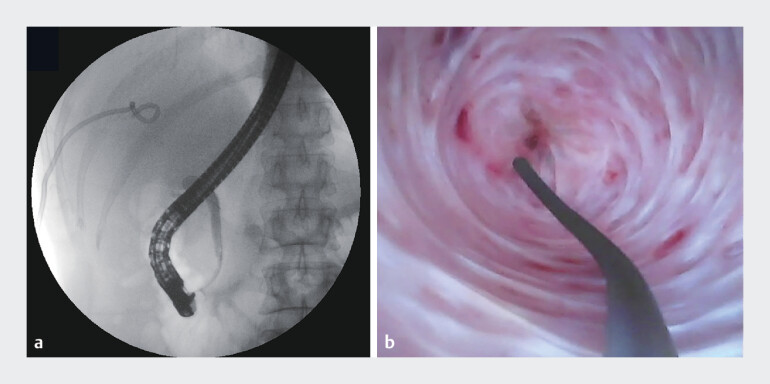
Endoscopic retrograde cholangiopancreatography (ERCP) technique failed. **a** Retrograde cholangiography revealed truncation of the common bile duct (CBD), with nonvisualization of the upper part of the CBD and intrahepatic bile duct. **b** Selective cannulation failed using direct cholangioscopy under ERCP.

**Fig. 3:**
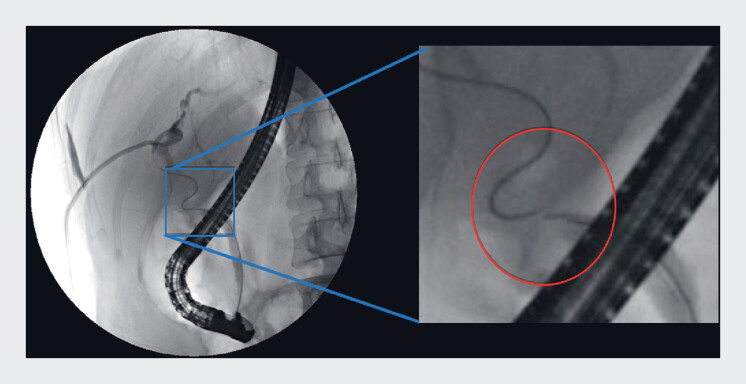
Classic rendezvous technique of percutaneous transhepatic biliary drainage and endoscopic retrograde cholangiopancreatography was unsuccessful in passing through the stricture.

**Fig. 4:**
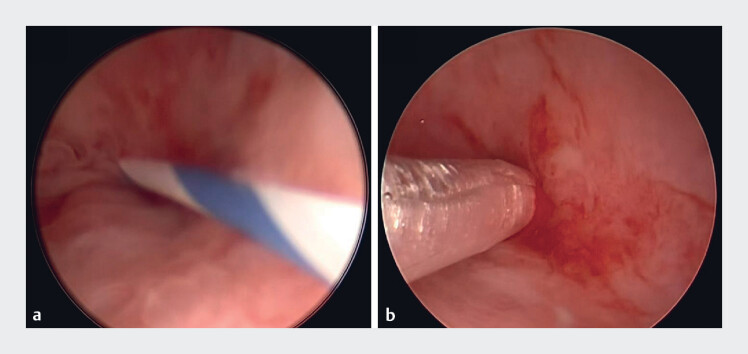
Percutaneous transhepatic cholangiography technique. **a** A rigid guidewire was inserted through the stricture under ultrasound guidance. **b** The stricture was dilated using a 6-mm balloon.

**Fig. 5:**
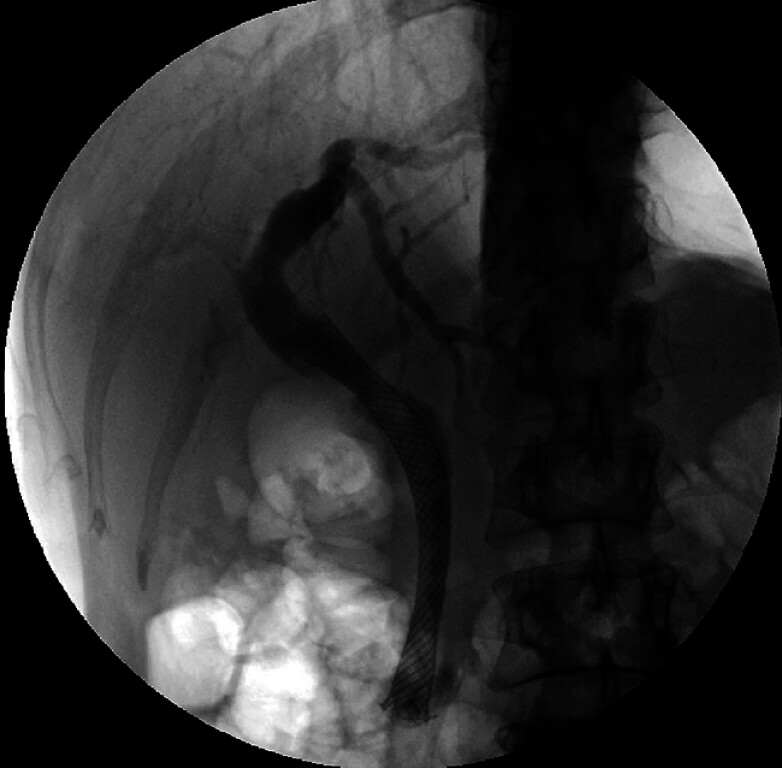
A 10 × 80 mm self-expanding covered metal stent was placed under endoscopic retrograde cholangiopancreatography.

Use of percutaneous transhepatic cholangiography for selective cannulation of anastomotic stricture following liver transplantation, after failure of endoscopic retrograde cholangiopancreatography and classic rendezvous technique.Video 1

The use of percutaneous transhepatic cholangiography for selective cannulation represents a novel approach to the therapeutic management of complex biliary stricture. This technique is particularly beneficial for cases where ERCP and classic rendezvous techniques have failed, allowing surgical treatment to be avoided.

Endoscopy_UCTN_Code_TTT_1AR_2AG

## Notes

Endoscopy E-Videos https://eref.thieme.de/e-videos
*E-Videos* is an open access online section of the journal *Endoscopy* , reporting on interesting cases and new techniques in gastroenterological endoscopy. All papers include a high-quality video and are published with a Creative Commons CC-BY license. Endoscopy E-Videos qualify for HINARI discounts and waivers and eligibility is automatically checked during the submission process. We grant 100% waivers to articles whose corresponding authors are based in Group A countries and 50% waivers to those who are based in Group B countries as classified by Research4Life (see: https://www.research4life.org/access/eligibility/ ). This section has its own submission website at https://mc.manuscriptcentral.com/e-videos .
